# Effect of atropine 0.01% on myopia control in children aged 6–13 years during the 2022 lockdown in Shanghai

**DOI:** 10.3389/fpubh.2023.1074272

**Published:** 2023-01-26

**Authors:** Manrong Yu, Lingli Jiang, Minjie Chen

**Affiliations:** ^1^Department of Ophthalmology, Eye and Ear, Nose, and Throat Hospital of Fudan University, Shanghai, China; ^2^Key Laboratory of Visual Impairment and Restoration of Shanghai, Fudan University, Shanghai, China; ^3^NHC Key Laboratory of Myopia, Fudan University, Shanghai, China; ^4^Key Laboratory of Myopia, Chinese Academy of Medical Science, Shanghai, China; ^5^Department of Ophthalmology, The Affiliated Wenling Hospital of Wenzhou Medical University, Wenling, China

**Keywords:** myopia control, atropine, COVID-19, Shanghai, myopia progression, quarantine, children, defocus incorporated multiple segments (DIMS)

## Abstract

**Purpose:**

To compare the myopic progression in children treated with 0. 01% atropine and those who discontinued atropine during the 2022-home quarantine in Shanghai.

**Methods:**

In this retrospective study, children aged 6–13 years with follow-up visits before (between January 2022 and February 2022) and after the lockdown (between July 2022 and August 2022) were included. Cycloplegic refraction and axial length (AL) were measured at both visits. The atropine group had continuous medication during the lockdown while the control group discontinued. The 0.01% atropine eyedrops were administered daily before bedtime. The types of spectacle lens were recorded: single vision (SV) spectacles or defocus incorporated multiple segments lenses (DIMS).

**Results:**

In total, 41 children (81 eyes) in the atropine group and 32 children (64 eyes) in the control group were enrolled. No significant difference was found in the demographic characteristics, spherical diopter, spherical equivalent (SE), AL, and follow-up time between the two groups before the lockdown in 2022 (all *p* > 0.1). After the home confinement, a greater myopia progression was observed in the control group (−0.46 ± 0.42 D) compared to atropine group (−0.26 ± 0.37 D; *p* = 0.0023). Axial elongation was also longer in the control group than that in children sustained with atropine (0.21 ± 0.17 vs. 0.13 ± 0.15 mm, *p* = 0.0035). Moreover, there was no significant change of spherical diopter and SE during lockdown in the atropine + DIMS combined subgroup (0.03 ± 0.033 D for spherical diopter, *p* = 0.7261 and 0.08 ± 0.27 D for SE, *p* = 0.2042, respectively). However, significant myopic shift was observed in the atropine + SV subgroup during the quarantine time (−0.31 ± 0.39 D for SE and 0.15 ± 0.16 mm for AL, both *p* < 0.001).

**Conclusion:**

Children treated with 0.01% atropine had slower myopia progression during the lockdown period in Shanghai compared with children discontinued. Moreover, the effect of atropine on myopic prevention can be strengthened with DIMS lenses.

## Introduction

The widespread prevalence of myopia makes it a major public health concern. By 2050, close to 50% of the world's population are expected to have myopia and with as much as 10% highly myopic (1). Meanwhile, the increasingly high prevalence of myopia and high myopia have been already witnessed in the past few decades (2). Moreover, early-onset myopia among preschoolers has become more prevalent in recent years in China (3), indicating longer duration of myopia progression and more likely to have high myopia in the future (4, 5). Consequently, the Chinese Ministry of Education issued the Comprehensive Plan to Prevent Nearsightedness among Children and Teenagers (CPPNCT) in aim to reduce the incidence of myopia and control myopic progression in China in 2018 (6). Though numerous items are included in the CPPNCT, increasing time outdoors and reducing near-work time are the key content (6). For example, mandatory outdoor time has already been implemented as part of myopia prevention programs in cities of mainland China.

However, the pandemic of coronavirus disease (COVID-19) in December 2019 has caused huge behavioral changes. Home confinement was imposed and children were forced to study *via* online platforms. As a result, significant progression in myopia has been evidenced in numerous reports during the lockdown (7–11). Considerable evidences are provided to support the association between the progression of myopia and an increase in near work or a reduction of outdoor activities during the COVID-19 home confinement (7, 8, 10). To make matters worse, in late February, 2022, another wave of COVID-19 infection rapidly appeared in Shanghai, China (12). Strict home confinement was applied again and the “home education” was implemented once more until the end of June.

Nowadays, low-concentration atropine eye drops is recommended and widely used to slow myopic progression (13–16). Though 0.05% atropine has been proved to be the optimal concentration for myopia control (15, 16), it is not commercially available in our country compared with 0.01% atropine which is clinically easy to prescribe in Shanghai. Only two clinical-based studies have observed that myopia could still progress during COVID-19 quarantine even in children treated with 0.01% atropine (17, 18). But neither study had a control group, indicating the same group of the children were analyzed and compared only in a temporal relation (17, 18). Thus it remains unknown if beneficial effect of 0.01% atropine could be achieved in comparison to the cessation of atropine treatment during the lockdown.

In this study, we analyzed the myopic progression among primary students treated with 0.01% atropine eye drops compared to those who discontinued during the 2022-home quarantine in Shanghai, aiming to explore the effect of 0.01% atropine in the prevention of myopia during the COVID-19 pandemic.

## Patients and methods

In this retrospective study, the medical records of children aged from 6 to 13 years old (Shanghai primary students from grades 1 to 7) who visited our refractive department clinic before (between January 2022 and February 2022) and after the lockdown (between July 2022 and August 2022) were reviewed. These students took online courses at home from March 13th until the end of the semester, they received comprehensive ophthalmic examinations within 2 weeks after 2 months lockdown. In total, the continuous subjects of 41 children (81 eyes) in the atropine group and 32 children (64 eyes) in the control group were included. All children were prescribed 0.01% atropine eye drops before 2022 according to the protocol which was when calculated myopic progression rate exceeded −0.75 diopter (D)/year (y). The atropine group had continuous medication during the lockdown, while the control group discontinued since the epidemic began in February 2022 due to the home quarantine and inconvenience to get the eyedrops. The 0.01% atropine drops were administered daily before bedtime. Parents were instructed to use the eye drops during clinic and would be asked whether their children used every day. The 0.01% atropine eyedrops were produced by adding 1 ml of 0.05% Kg/L atropine sulfate (atropine sulfate injection, Hubei Xinghua Pharmaceutical Co., Ltd., China) to 4 ml of polyethylene glycol eye drops (Systane ULTRA, Alcon Laboratories, Inc., USA) by the Pharmaceutical Department of Eye & ENT Hospital (19). To further analyze the data, we classified children in the atropine group into single vision (SV) spectacle lenses subgroup and defocus incorporated multiple segments (DIMS, MiYOSMART, manufactured by HOYA Corporation, Japan) lenses subgroup. Children were asked to wear the spectacles in full-time mode, for no less than 12 hours per day. This study was conducted in accordance with the Declaration of Helsinki and the protocol was approved by the Institutional Review Board of the Eye and ENT Hospital of Fudan University. Parents or guardians of patients aged < 18 years provided written informed consent.

All children received comprehensive ophthalmic examinations. The best-corrected visual acuity in both eyes of all children was not less than 0.0 Log MAR. Subjects with any other eye diseases, injury and history of orthokeratology or concentric contact lenses were excluded from the study.

Cycloplegic refraction was measured at both visits. Each subject was administered five drops of compound tropicamide eyedrops (0.5% tropicamide and 0.5% phenylephrine eyedrops; Univision, China) with a 5-min interval. Three readings of spherocylindrical auto-refraction (KR-8800, Topcon Corporation, Tokyo, Japan) were taken and averaged 30 min after the last eyedrop. Subjective refraction was then performed by an experienced optometrist. IOL Master (Carl Zeiss Meditec AG, Jena, Germany) was used to measure axial length (AL). Five repeated measurements were taken and averaged before cycloplegia.

### Statistical analysis

All statistical analyses were performed using the Stata 14.0 (Stata Corp., College Station, TX, USA). Both eyes were included in the analysis. The spherical equivalent (SE) was calculated as spherical power plus half-negative cylinder power. A paired *t*-test or matched-pairs signed-rank test was used to analyze the time course difference in both groups. Between-group differences were checked with the *t*-test or Wilcoxon rank-sum test. Numeration data were compared between the two groups using χ^2^ tests. The statistical significance threshold was set at *p* = 0.05.

## Results

There was no significant difference in terms of gender and age distribution between the two groups, ranging from 6 to 13 years (Table 1). Before the lockdown, the mean spherical diopter was −1.53 ± 1.30 and −1.26 ± 1.29 D in the atropine group and control group, respectively (*p* = 0.2106). SE and AL were also comparable between the two groups. There were nine subjects wearing DIMS lenses in the atropine group compared to four subjects in the control group (*p* = 0.295).

**Table 1:** Baseline general characteristics of all participants.

	**Atropine group** **(*n* = 41, 81 eyes)**	**Control group** **(*n* = 32, 64 eyes)**	***p-*Values**
Age (year)	9.39 ± 1.58	9.41 ± 1.93	0.9690
Female, no. (%)	41.46%	31.25%	0.370
DIMS lenses, no. (%)	21.95%	12.50%	0.295
Follow-ups (month)	5.13 ± 0.10	5.20 ± 0.10	0.6280
Spherical diopter (D)	−1.53 ± 1.30	−1.26 ± 1.29	0.2106
Spherical equivalent (D)	−1.75 ± 1.36	−1.45 ± 1.36	0.1890
AL (mm)	24.47 ± 0.93	24.24 ± 0.83	0.1198

DIMS, defocus incorporated multiple segments; D, diopter; AL, axial length.

**Figure 1:**
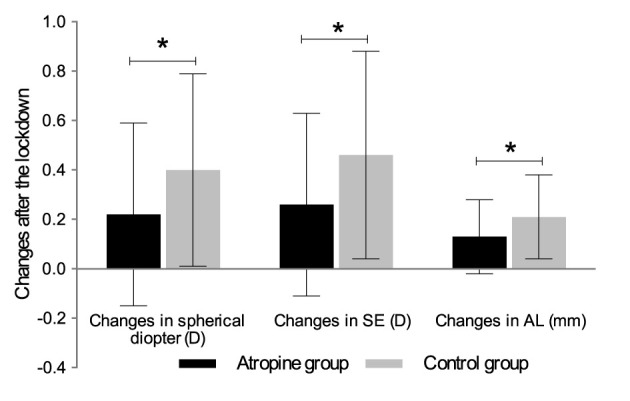
Changes of spherical diopter, SE and AL in atropine and control group after the 2022-lockdown in Shanghai. *Significant difference between the two groups. SE, spherical equivalent; AL, axial length.

Myopic shift was found in both groups after the lockdown (all *p* < 0.001). During the lockdown, the change in SE in the control group was significantly greater than that in the atropine group (*p* = 0.0023), with an average myopic shift of −0.46 ± 0.42 and −0.26 ± 0.37 D in the control group and atropine group, respectively. Similar results were observed in spherical diopters (*p* = 0.0053; Figure 1). A marked increase in AL was seen in both groups with 0.13 ± 0.15 mm in the atropine group and 0.21 ± 0.17 mm in the control group during the lockdown (both *p* < 0.05). Greater changes in AL were seen in children who discontinued medication during the 2022 pandemic (*p* = 0.0035).

### Additive effect of DIMS lenses

**Figure 2:**
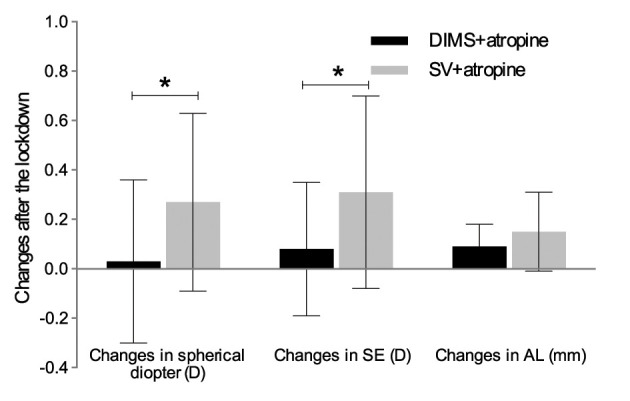
Changes of spherical diopter, SE and AL in DIMS + atropine and SV + atropine group after the 2022-lockdown in Shanghai. *Significant difference between the two groups. SE, spherical equivalent; AL, axial length; DIMS, defocus incorporated multiple segments lenses; SV, single vision spectacle lenses.

The additive effect of DIMS spectacle lenses on myopia control was also studied. Children sustained with atropine in the current study were classified into two subgroups according to whether wearing DIMS lenses or SV spectacles. Though age and gender distribution were comparable between the two populations, children wearing DIMS lenses had more myopia as well as AL (Table 2). After home confinement, a myopic progression of −0.27 ± 0.36 D was found in SV + atropine subgroup (*p* < 0.001), while no significant myopia shift was observed in DIMS + atropine group (Figure 2; 0.03 ± 0.033 D for spherical diopter, *p* = 0.7261 and 0.08 ± 0.27 D for SE, *p* = 0.2042, respectively). The mean change of AL was 0.15 ± 0.16 mm in the SV + atropine monotherapy group during the home quarantine, which was 0.09 ± 0.09 mm in the DIMS + atropine combined therapy subjects.

**Table 2:** Demographic characteristics and ocular parameters based on the types of spectacle lenses wearing.

	**SV + atropine** **(*n* = 32, 63 eyes)**	**DIMS + atropine** **(*n* = 9, 18 eyes)**	***p-*Values**
Female, no. (%)	40.63%	44.44%	0.8370
Age (year)	9.44 ± 1.63	9.22 ± 1.48	0.7228
Follow-ups (month)	5.16 ± 0.12	5.06 ± 0.15	0.6720
Spherical diopter (D)	−1.32 ± 1.26	−2.26 ± 1.19	0.0060
Spherical equivalent (D)	−1.54 ± 1.33	−2.49 ± 1.25	0.0078
AL (mm)	24.30 ± 0.93	25.07 ± 0.66	0.0017

SV, single vision spectacle lenses; DIMS, defocus incorporated multiple segments lenses; D, diopter; AL, axial length.

## Discussion

Nowadays, as the pandemic of COVID-19 continues and the Chinese government enacts strict stay-at-home quarantine policies, studying at home during the pandemic is inevitable, accompanied by more digital screen time and less outdoor activities. It is urgent to find effective and generalizable methods to prevent and control myopia in children. In the current study, during the 2022-home-quarantine period in Shanghai, less myopia progression was found in 6-to 13-year-old students who received 0.01% atropine compared to children who discontinued. Moreover, the combination of 0.01% atropine and DIMS lenses offered benefit in controlling myopia during the pandemic.

Numerous studies demonstrated myopia progression during the study-at-home period was higher than the highest myopia progression during the period before COVID-19 (7–11). Most studies focused on the progression rate from 2019 to 2020, when this pandemic first broke out. However, all the children in Shanghai experienced quarantine again in 2022. One research in Shanghai reported that myopic progression in children aged 7–12 years was −0.98 ± 0.52 D during the 5.4 months of the first pandemic (7), which was nearly twice greater than that in current study of −0.46 ± 0.42 D in the control group during the 2022 pandemic of 5.2 months. Another study in Wenzhou found an average −0.343D myopia progression at 6-month for all schoolchildren after the first COVID-19 quarantine (20), consistent with the data identified in Chongqing, China (21). We speculate that this difference could be due to a number of factors. First of all, differences in optometric methods, inclusion criteria and educational environment may account for part of the discrepancy. Secondly, myopia development in Chinese children during study-at-home attracted much attention since the pandemic in 2019. Public awareness and educational campaigns focusing on preventive strategies of myopia have been gradually promoted, including avoiding prolonged near work activities, room lighting, writing posture, interval break after 20 min of screen time and preferred larger screen (22). Therefore, it is reasonable that these forced behavioral changes due to the 2022 pandemic may have an impact on children's refractive status.

Against the rapid and widespread development of myopia, a series of interventions for myopia control in children have reached high-level evidence, among which low concentration atropine is an effective one (13–16). This study compared the myopic progression in children treated with 0.01% atropine or not during the 2022 quarantine, and found the myopic progression decelerated in children with continuous medication since the outbreak of the pandemic in February 2022, though two previous studies showed that 0.01% atropine had little effect on myopic progression during the lockdown (17, 18). For one thing, the refractive data was collected in the same group at different ages in previous studies (Longitudinal cohort), indicating that the data was analyzed and compared in a time course (17, 18). In another word, neither previous studies included an age-matched control group, which was complementarily designed in the present study. It is well-acknowledged that there is a natural progression of axial elongation among children. The design of this study eliminated the confounding effect of natural myopia progression with increasing age and therefore might reflect the true effect of atropine on myopia progression during the lockdown. For another, younger age of children in Yum et al. (18) study located in South Korean and relatively small sample size (14 children) in Erdinest et al. (17) study located in Israel may also interpret their insignificant results. Besides, children in the current control group were also applied with 0.01% atropine before the pandemic in 2022. Recently, a cross-over trial suggested that there is no rebound effect after cessation of 0.01% atropine eyedrops in preventing myopia progression (23). Thus in the present study, children in control group experienced natural development of myopia and had more myopic shift and axial elongation in contrast with the atropine group.

Though interventions for controlling myopia progression were widely applied, neither of them could achieved a 100% success (24). Subsequently, several studies have investigated the effect of combination therapy, and revealed that the addition of orthokeratology and low-dose atropine was better than orthokeratology alone in myopia control (19, 24–26). To our knowledge, this is the first study showing adjunctive effect of DIMS lenses and 0.01% atropine in controlling myopic progression in 6-to 13-year-old students during the home quarantine period in 2022. The specially designed DIMS lenses has been proved to have greater effect in myopia control compared to SV lenses through incorporating myopic defocus (27). Furthermore, the effect of DIMS spectacles was also proved to significantly prevent myopia progression compared with SV lenses treatment during the lockdown period (28). Consistent with the previous study, negligible increments of 0.08 ± 0.27 D in SE and 0.09 ± 0.09 mm in AL were evidenced in DIMS and atropine combined therapy compared with significant myopic progression in children treated with atropine alone in the present study. As a kind of frame glasses, DIMS lenses are convenient for children, making it easy to be promoted and applied. The complementary effect of DIMS lenses and atropine observed here enlightens the synergistic treatments in myopia control in the future. Though DIMS lenses were also used in control group here, the small sample size (four children) prevented us from analyzing its effect any further.

However, this study has several limitations. Firstly, the sample size was relatively small, so both eyes were included in the analysis which could have created potential bias. Secondly, data about time spent each day on learning, on different types of digital screen devices (including mobile phone, tablet, television, and projector) as well as outdoor activities were not collected. Multiple studies have concluded that development of myopia accelerated during pandemic due to the excessive time spent on digital screen devices for online learning and less time spent on outdoor activities (8, 29). Without data of those behavioral changes during the lockdown, the associated factors influencing the myopic progression could not be interpreted. Thirdly, this study did not collect data on rate of myopic progression before the 2022-pandemic, which made the investigation less comprehensive and convincing.

In summary, pharmaceutical treatment with 0.01% atropine was significantly associated with slower myopia progression compared to children who experienced cessation of therapy during 2022 lockdown in Shanghai. In addition, better performance was observed in synergistic efforts of DIMS spectacles and 0.01% atropine in myopic prevention. The findings from our data may help better to adjust the behaviors to prevent myopia and the progression in the future, especially during the quarantine period of COVID-19. This study could pave wave for better understanding regarding the efficacy of current myopia control measures by helping to design a larger and multicenter study.

## Data availability statement

The original contributions presented in the study are included in the article/supplementary material, further inquiries can be directed to the corresponding author.

## Ethics statement

The studies involving human participants were reviewed and approved by Institutional Review Board of the Eye and ENT Hospital of Fudan University. Written informed consent to participate in this study was provided by the participants' legal guardian/next of kin.

## Author contributions

MY was responsible for collecting and analyzing data, interpreting results, and writing the abstract and the paper. LJ contributed to reviewing of the data, interpreting results, and writing the paper, tables, and figures. MC contributed to the design of the study, review, and feedback on the paper. All authors contributed to the article and approved the submitted version.
